# Human Umbilical Mesenchymal Stem Cells Display Therapeutic Potential in Rheumatoid Arthritis by Regulating Interactions Between Immunity and Gut Microbiota *via* the Aryl Hydrocarbon Receptor

**DOI:** 10.3389/fcell.2020.00131

**Published:** 2020-03-13

**Authors:** Xiaoya Li, Cheng Lu, Danping Fan, Xiangchen Lu, Ya Xia, Hongyan Zhao, Huihui Xu, Yongliang Zhu, Jingtao Li, Honglin Liu, Cheng Xiao

**Affiliations:** ^1^Department of Emergency, China-Japan Friendship Hospital, Beijing, China; ^2^Institute of Clinical Medical Sciences, China-Japan Friendship Hospital, Beijing, China; ^3^Graduate School of Peking Union Medical College, Chinese Academy of Medical Sciences/Peking Union Medical College, Beijing, China; ^4^Institute of Basic Research in Clinical Medicine, China Academy of Chinese Medical Sciences, Beijing, China; ^5^School of Traditional Chinese Medicine, Beijing University of Chinese Medicine, Beijing, China; ^6^Beijing Key Laboratory of Research of Chinese Medicine on Prevention and Treatment for Major Diseases, Experimental Research Center, China Academy of Chinese Medical Sciences, Beijing, China; ^7^Department of Plant and Microbial Biology, University of California, Berkeley, Berkeley, CA, United States; ^8^Department of Gastroenterology, China-Japan Friendship Hospital, Beijing, China

**Keywords:** human umbilical mesenchymal stem cells, rheumatoid arthritis, host immunity, gut microbiota, aryl hydrocarbon receptor

## Abstract

**Background:**

Rheumatoid arthritis (RA) is an autoimmune disease that may be associated with gut microbiota *via* the aryl hydrocarbon receptor (AhR). Human umbilical mesenchymal stem cells (HUMSCs) have therapeutic potential against RA, but the underlying mechanism has not been fully elucidated. The purpose of this study was to explore the mechanism of action of HUMSCs in rats with collagen-induced arthritis (CIA).

**Method:**

HUMSCs (1 × 10^6^) were transplanted into each rat with CIA. The tissue localization of HUMSCs and the therapeutic effects in the ankles were assessed. The immune status and expression of immune-related genes and proteins in related lymphoid tissues were subsequently tested. Furthermore, the levels of immune-related factors in serum and the changes in gut microbiota in the ileum were detected, and the levels of indole and their derivatives in plasma and the levels of AhR in the ileum were evaluated.

**Results:**

HUMSCs homed to the popliteal lymph node (PLN), mesenteric lymph node (MLN), ankle cartilage, and ileum mucosa in rats with CIA. The transplantation of HUMSCs reduced the pathology scores and the degree of bone damage in the ankles. The immune status of T regulatory cells (Tregs) and T helper (Th)17 cells and the gene expression levels of interleukin (IL)-10, transforming growth factor (TGF)-β*1*, and IL-17A were altered in the PLN, which is the lymph tissue closest to the nidus, and the MLN, which is one of the gut-associated lymphoid tissues (GALTs). The proportion and function of B cells, Tregs, and Th17 cells were regulated in other GALTs, namely, Peyer’s patches and the lamina propria. The gene expression of TGF-β*1* and IL-17A and protein expression of IL-10, TGF-β*1*, IL-17A, IL-22, and immunoglobulin A (IgA) were modulated in the ileum, and the serum levels of IL-10, TGF-β1, IL-17A, IL-1β, and tumor necrosis factor (TNF)-α were regulated in the rats with CIA. The relative abundances of the genera *Bacteroides* and *Bacillus* were increased in the HUMSCs-treated rat with CIA; in addition, the levels of indole, indoleacetic acid, and indole-3-lactic acid were consistently upregulated, and this upregulation was accompanied by increases in AhR gene and protein expression.

**Conclusion:**

Our study demonstrates that HUMSCs play a therapeutic role in rats with CIA by regulating the interactions between host immunity and gut microbiota *via* the AhR.

## Introduction

Rheumatoid arthritis (RA) is a systemic, inflammatory, and autoimmune disease characterized by immune disorders and bone dysmetabolism that ultimately leads to functional disability. RA, one of the most common autoimmune diseases, has an incidence of 0.5%–1% worldwide and is three times more common in women than in men (Smolen et al., 2016). Previous studies have revealed that the complex interplay among genotype, environmental triggers, and chance is involved in the pathogenesis and progression of RA (McInnes and Schett, 2011). In addition, in-depth research in microbiota has gradually revealed that the bidirectional influence between gut microbiota/their metabolites and host immunity might be related to RA, and alterations in the gut microbiota have been found to be involved in improving RA symptoms (Wu X. et al., 2016). RA is correlated with the inflammatory response mediated by CD4^+^ T helper 1 (Th1) lymphocytes, T helper 17 (Th17) lymphocytes, and an imbalance between Th17 lymphocytes and T regulatory cells (Tregs) (Noack and Miossec, 2014).

Stem cells are cells with multidifferentiation capacity that exert immunomodulatory effects by modulating T and B cell proliferation and differentiation, dendritic cell maturation, and natural killer (NK) cell activity (Naik et al., 2018). In addition to their self-renewal ability, human umbilical mesenchymal stem cells (HUMSCs) exhibit minimal immune rejection and can be used for allogeneic transplantation (Ma et al., 2019). The abovementioned characteristics indicate the potential of using HUMSCs to treat autoimmune diseases (ADs), and this finding has been verified (Uccelli et al., 2008). Specifically, the use of HUMSCs in RA has shown promising results in clinical trials (Shadmanfar et al., 2018; Ghoryani et al., 2019). Basic studies have also suggested that HUMSCs can alleviate collagen-induced arthritis (CIA) partly by regulating Tregs and Th17 cells (Liu et al., 2010; Tong et al., 2015; Ma et al., 2019), but further research on the molecular mechanisms and interconnections between these different mechanisms remains rare.

The aryl hydrocarbon receptor (AhR) is a cytoplasmic receptor that responds to multiple exogenous and endogenous compounds from the diet, host metabolites, and the gut microbiota (Shinde and McGaha, 2018). Tryptophan metabolites are the main ligands of the gut microbiota (Gao et al., 2018). An increasing number of studies have shown that the AhR plays an indispensable role in the regulation of innate and adaptive immunity, including the regulation of the differentiation and function of Tregs and Th17 cells (Rothhammer and Quintana, 2019). Therefore, the AhR plays a key role in connecting immunity to the microenvironment. Although drugs that regulate Tregs and Th17 cells in CIA reportedly function through the AhR and the interaction between the AhR and the gut microbiota affects the disease status, no interaction among the gut microbiota, the AhR, Tregs/Th17 cells, and HUMSCs has been detected.

In this study, we detected the suppressive effect of HUMSCs on the differentiation of osteoclasts (OCs) in CIA. We also demonstrated that HUMSCs can regulate the immune status of the lymph tissue closest to the nidus—the popliteal lymph node (PLN). More importantly, the study showed that the immune status of gut-associated lymphoid tissues (GALTs) and the gut microbiota in the ileum were also altered by HUMSCs. In addition, the possible mechanisms underlying these effects were explored.

## Materials and Methods

### Isolation and Culture of Human Umbilical Mesenchymal Stem Cells and Phenotype Identification

This study was approved by the Research Ethics Committee at the China-Japan Friendship Hospital (2019-124-K86). Fresh umbilical cord samples were obtained from the Obstetrics Department at the China-Japan Friendship Hospital after normal spontaneous full-term delivery, and written informed consent was provided by the mother. The umbilical tissue was transferred into Hanks’ balanced salt solution (HBSS) (Gibco BRL Life Technologies, Grand Island, NY, United States) to remove aggregation and blood vessels. The remaining tissue was then dissected into cubes approximately 0.5 cm^3^ in size and incubated with 0.25% trypsin (Gibco) in an incubator for 15 min. The tissue was transferred to culture vessels containing high-glucose Dulbecco’s modified Eagle’s medium (DMEM; Gibco) with 10% fetal bovine serum (FBS; Gibco) and antibiotics (100 IU/ml penicillin, 100 μg/ml streptomycin; Gibco) at 37°C in 5% CO_2_ and left undisturbed for 2 to 4 weeks to allow the migration of HUMSCs from the tissues. Once many cells surrounded the tissue, the cells were refed and passaged. After the third passage, the cells were harvested for phenotype identification through staining with antibodies against CD34, CD45, CD11b, CD19, HLA-DR, CD73, CD90, and CD105 (BD Pharmingen, San Diego, CA, United States) and analyzed with a FACS Canto II flow cytometer (FACS Canto II, Becton, Dickinson, Co., Franklin Lakes, NJ, United States). HUMSCs after the third to fifth passages were used for the experiments.

### Induction of Arthritis and Arthritis Assessment

Forty male Sprague-Dawley rats (190 ± 10 g) were purchased from the National Institutes for Food and Drug Control [animal license number: SCXK (Beijing) 2014-0013]. The rats were maintained in a specific pathogen-free animal laboratory with an environment with a constant temperature of 23°C (± 2°C) and an alternating 12-h light/12-h dark cycle at the Experimental Animal Center of the Institute of Clinical Medical Sciences, China-Japan Friendship Hospital [Experiment Animal Center license number: SYXK (Beijing) 2016-0043]. Two to three rats were housed in each cage with free access to standard rodent chow and water. All the experimental procedures were examined and approved by the Institute of Clinical Medical Sciences, China-Japan Friendship Hospital, Beijing, China (No. 180207).

After the rats were subjected to adjustable feeding for 7 days, arthritis was induced as previously described (Zhao et al., 2018). Briefly, the rats were immunized on day 0 with 100 μg of bovine type II collagen (Chondrex, Inc., Redmond, WA, United States) emulsified in isopyknic incomplete Freund’s adjuvant (Chondrex) *via* intradermal injection at one side of the base of the tail while avoiding the tail vein and administered booster immunization with the same preparation on day 7 on the other side of the base of the tail. The arthritis severity was examined every 3 days starting on day 1 and expressed through an arthritic index (AI) ranging from 0 to 4 points each hind leg according to conventional criteria (Zhao et al., 2018) as follows: 0 = no change, 1 = red or slight swelling, 2 = mild swelling, 3 = pronounced swelling, 4 = limb deformity and inability.

### Experimental Groups and Treatments

After the onset of arthritis, the CIA model rats with no change in their AI score were excluded, and the remaining rats were randomly divided into the following three groups and started to undergo treatment on day 10: (1) normal control rats (Control group), (2) rats with CIA (CIA group), (3) CIA rats with methotrexate (MTX, Xinyi, Shanghai, China) (MTX group), and (4) CIA rats transplanted with HUMSCs (HUMSCs group). The rats in the HUMSCs group were administered 1 × 10^6^ HUMSCs suspended in 200 μl of a 0.9% NaCl solution, and the rats belonging to the other groups were administered an equal solution of 0.9% NaCl solution *via* tail vein injection. The rats in MTX group were intragastrically administered MTX (1.5 mg/kg twice a week), and the rats in the control, CIA, and HUMSCs groups were treated with the same volume of pure water as MTX group.

### Immunohistochemistry and Immunofluorescence

The rats were sacrificed on day 38 (after 28 days of treatment). For immunohistochemistry (IHC) assessment, paraffin-embedded sections (5-μm-thick) of the spleen, PLN, mesenteric lymph node (MLN), ileum, and knee joints after decalcification with 10% EDTA-Na_2_ were heated in a water bath for antigen retrieval using citrate buffer (pH 6.0) or ethylenediaminetetraacetic acid (EDTA; pH 9.0) depending on the primary antibody. The sections were then incubated with 3% hydrogen peroxide (H_2_O_2_) for 15 min away from light. The ileum sections were incubated with the following primary antibodies: rabbit anti-rat interleukin (IL)-10 (Abcam, Cambridge, MA, United States; 1:400), rabbit anti-rat transforming growth factor (TGF)-β*1* (Abcam; 1:100), rabbit anti-rat IL-17A (Absin, Shanghai, China; 1:100), rabbit anti-rat IL-22 (Absin; 1:500), goat anti-rat immunoglobulin A (IgA; Abcam; 1:400), and rabbit anti-rat AhR (Proteintech, Rosemont, IL, United States; 1:200). Sections of the spleen, PLN, MLN, ileum, and knee joints of the HUMSCs group were incubated with primary mouse anti-human nuclear mitotic apparatus (NuMA) (Santa Cruz Biotechnology; 1:200) (a human cell-specific nuclear antigen) in antibody diluent overnight at 4°C and then with horseradish peroxidase (HRP)-linked species-specific antibodies for 20 min. The sections were subsequently stained with 3,3′-diaminobenzidine (DAB) and counterstained with hematoxylin at room temperature. Images were randomly captured using a ZEISS Axio Observer 3 microscope, and Image Pro-Plus was used to determine the positive staining area and the integral optical density of each index, which was calculated with the following formula: mean optical density = integral optical density/positive staining area. The immunofluorescence (IF) experiments involved the use of primary mouse anti-human NuMA antibody and no incubation with secondary antibody. Specifically, in the IF experiments, the samples were incubated with Alexa Fluor 555-conjugated species-specific antibodies (Cell Signaling Technology, Boston, MA, United States) for 1 h at room temperature in the dark and then sealed with mounting medium containing 4’,6-diamidino-2-phenylindole (DAPI; Invitrogen, Carlsbad, CA, United States). Images were captured randomly using a laser scanning confocal microscope (Zeiss, LSM 800).

### Histopathological Evaluation and Tartrate-Resistant Acid Phosphatase Staining of Ankle Bones

The left ankle bones were decalcified and used to prepare 5-μm-thick slices, and the slices were then stained with Hematoxylin-Eosin staining (H&E). The mean inflammation score was assessed microscopically on a scale of 0–3 according to the degree of lymphocyte infiltration, synovial hyperplasia, pannus, cartilage damage, and joint destruction (0 = normal, 1 = weak, 2 = moderate, and 3 = severe). The OCs of the ankle joints were counted by staining slices of the ankle samples with tartrate-resistant acid phosphatase (TRACP; Sigma, St. Louis, MO, United States) and were identified by the presence of multinucleated cells that stained positive for TRACP and contained at least three nuclei.

### Micro-CT Analysis

A Skyscan 1174 micro-CT scanner (Bruker, Belgium) was used to scan the left ankle joints and paws of the rats, and three-dimensional (3D) images were reconstructed with the corresponding software, N-Recon, to obtain the 3D model. The corresponding CT-AN software was then used for 3D analysis to detect the following indices: bone volume (BV), bone surface (BS), and ratio of bone surface to bone volume (BS/BV).

### Flow Cytometry

PLN, MLN, Peyer’s patch (PP), and lamina propria lymphocyte (LPL) were evaluated according to the manufacturer’s recommended protocol. Briefly, the lymph nodes and PPs of the four groups were isolated, placed in sterile normal saline solution and then ground with a 300-mesh sieve. The cell suspension was centrifuged, suspended in RPMI medium containing 10% FBS and filtered through 70-μm cell sieve. LPL was isolated as previously reported (Xiao et al., 2006), and antibodies against CD4^+^ and IL-17^+^ were then used to identify Th17 cells. However, cytoplasmic IL-17 was easily secreted into the extracellular space, and the concentration of IL-17 was low. Therefore, a cell stimulation cocktail with protein transport inhibitors (eBioscience, San Diego, CA, United States) was used for IL-17 stimulation overnight at 37°C prior to antibody staining. Antibodies against CD4^+^, CD25^+^, and Foxp3^+^ were used to stain Tregs, and antibodies against Foxp3 were added to the cells after fixation and permeabilization using a Foxp3/Transcription Factor Staining Buffer Set (eBioscience). B cells were identified as CD45RA^+^ and CD3^–^ cells. The Tregs and Th17 cells in the PLN, MLN, and PP and among the LPL and the B cells in the MLN and PP and among the LPL were analyzed using a FACS Canto II flow cytometer.

### Quantitative Real-Time PCR

The mRNA expression of IL-10, TGF-β1, and IL-17A in both the PLN and MLN and the mRNA expression of IL-10, TGF-β1, IL-17A, and AhR in the ileum were evaluated by quantitative real-time PCR (Q-PCR). Total RNA was extracted from the four groups using the TRIzol reagent (Invitrogen) according to the manufacturer’s recommended procedure, and gDNA elimination and cDNA synthesis were then performed using a PrimeScript RT Reagent Kit with gDNA Eraser (TaKaRa, Tokyo, Japan). PCR was performed using SYBR Premix Ex Taq (Tli RNaseH Plus) (TaKaRa) with a Quant Studio 5 Real-Time PCR System (Thermo Fisher Scientific, Waltham, MA, United States). Amplification was performed using the following steps: initial denaturation at 95°C for 30 s and 40 cycles of 95°C for 15 s and 60°C for 34 s. All the experiments were performed in duplicate, and the relative mRNA expression levels were determined by the 2^–Δ^ ^Δ^ ^Ct^ method. The primers used in this analysis are shown in Table 1.

**TABLE 1:** Specific primers used for PCR.

Gene	Forward primer	Revised primer
IL-10	5′-GATCCAGAGATCTTAGCTA-3′	5 ′-CTGAGGTATCAGAGGTAA-3′
TGF-β1	5′-ATCGACATGGAGCTGGTGA-3′	5′-TTGGCATGGTAGCCCTTGG-3′
IL-17A	5′-GGAGAATTCCATCCATGTGC-3′	5′-CAGAGTCCAGGGTGAAGTGG-3′
AhR	5′-CGCGGGCACCATGAGCAG-3′	5′-CTGTAACAAGAACTCTCC-3′
β-Actin	5′-CACCCGCGAGTACAACCTTC-3′	5′-CCCATACCCACCATCACACC-3′

*IL, interleukin; TGF, transforming growth factor; AhR, aryl hydrocarbon receptor.*

### Quantitation of Serum Cytokines

The quantifications were performed according to the manufacturer’s instructions. The serum levels of the inflammatory and immune-related factors IL-10, TGF-β1, IL-17A, IL-1β, and tumor necrosis factor (TNF)-α were determined by Shanghai Laizee Biotech Co., Ltd. (China) using serological Luminex multiplex cytokine analysis technology. A Bio-Plex 200 system (Bio-Rad Laboratories, Hercules, CA, United States) was used to acquire the data.

### Microbiota Analysis by 16S rRNA Gene Sequencing

The ileum intestinal contents were harvested, immediately shock-frozen in liquid nitrogen, and then transferred to −80°C. A QIAamp PowerFecal DNA Kit (Qiagen, Valencia, CA, United States) was used to extract microbial DNA from the digesta according to the manufacturer’s instructions. The V3–V4 region of 16S rRNA was amplified by PCR using the 16S rRNA universal primer. The PCR products were purified using a QIAquick Gel Extraction Kit (Qiagen) and quantified with the Nanodrop and Qubit systems, respectively. The purified products underwent PE250 sequencing using HiSeq 2500 according to standard protocols. After quality inspection and sequence splicing, effective sequences were obtained for further analysis. Using the Greengenes database as a reference, sequences with 97% similarity into operational taxonomic units (OTUs) were classified using the usearch61 clustering method in QIIME (v1.9.1) software, and the OTUs were annotated according to reference taxonomy in the Ribosomal Database Project (RDP) database to obtain the taxonomic assignment. The alpha diversity was analyzed using QIIME and R to obtain indices of richness (the Chao1 diversity index) and diversity (the Shannon or Simpson index). The relative abundance was estimated in terms of the phylum, genus, and species. The intestinal microbial community was analyzed *via* partial least square discriminant analysis (PLS-DA). The differences between groups at different levels of taxonomy were obtained by Lefse analyses.

### Detection of Indole and Its Derivatives in Plasma

The levels of indole, indoleacetic acid (IAA), and indole-3-lactic acid (ILA) in the plasma were detected by liquid chromatography-tandem mass spectrometry (LC-MS/MS). A standard stock solution was gradient diluted into working standard solutions with different target concentrations. Subsequently, 80 μl of the plasma samples or working standard solutions was mixed with 240 μl of precipitating agent containing 2 μg/ml 3-methyl-d3-indole. The admixture was vortexed for 5 min and then centrifuged at 13,200 rpm and 4°C for 10 min, and the supernatant was the final solution used in the LC-MS/MS system.

Chromatographic separation was achieved with an Acquity UPLC BEH C8 (1.7 μm, 2.1 mm × 100 mm) column (Waters, Milford, MA, United States) using a mobile phase of (A) 0.01% formic acid and (B) acetonitrile at a flow rate of 0.3 ml/min. The gradient elution program was as follows: 0–2.0 min, 10% B; 3.0–4.0 min, 30% B; 5.0–7.0 min, 100% B; and 7.1–9.0 min, 10% B.

MS detection was performed using an Xevo TQ-S micro spectrometer (Waters) equipped with an electrospray ionization (ESI) source in the multiple reaction monitoring (MRM) mode. The optimized MS parameters were as follows: capillary voltage, 3,000.00 V; desolvation temperature, 550°C; cone gas flow, 10 L/h; desolvation gas flow, 10.00 L/h; and target column temperature, 45°C. TargetLynx (Waters) was used for data acquisition and quantitation.

### Statistical Analysis

The statistical analyses were performed with SPSS statistics 22.0 software. The significance of the differences was determined by unpaired *t*-tests (Mann–Whitney) or one-way analysis of variance (ANOVA) followed by the least significant difference (LSD) test. The non-parametric Kruskal–Wallis and Mann–Whitney *U* tests were used if the data were not normally distributed. Each data point was expressed as the mean ± *SEM* or median [interquartile range (IQR)]. *P* < 0.05 indicated significance.

## Results

### Identification of Human Umbilical Mesenchymal Stem Cells

**FIGURE 1:**
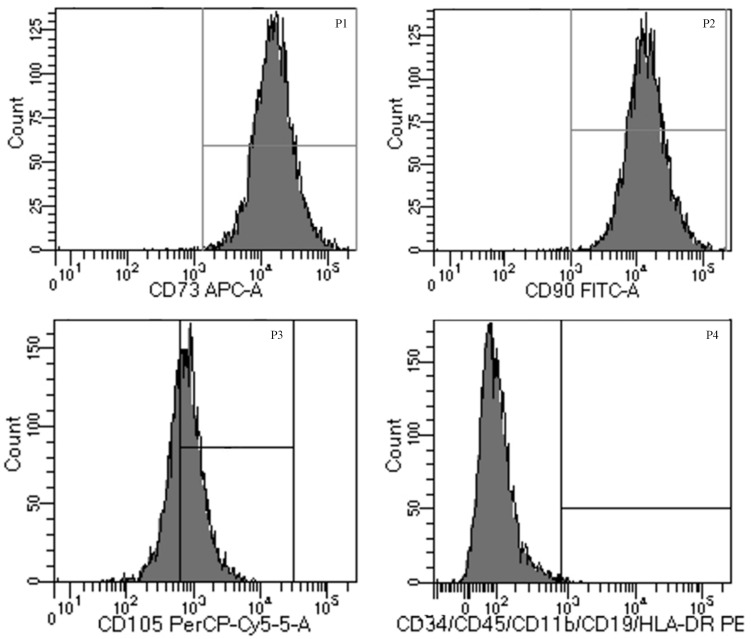
Characteristics of human umbilical mesenchymal stem cells (HUMSCs). The cells were labeled with APC, Allophycocyanin; FITC, Fluoresein Isothiocyanate; PerCP-cy5-5, Peridinin-ChlorophyII-Protein Complex-Cyanin 5-5; PE, Phycoerythrin-conjugated anti-HUMSC surface markers and analyzed by flow cytometry.

Cells were successfully isolated and cultured from fresh umbilical cord samples. HUMSCs were identified by harvesting cells at the third passage and analyzing them by flow cytometry. The cells were positive for the expression of CD105, CD90, and CD73 but negative for the expression of HLA-DR, CD11b, CD19, CD34, and CD45 (Figure 1). The results showed that the cells obtained from fresh umbilical cord were HUMSCs and could be used for subsequent experiments.

### Effects of Human Umbilical Mesenchymal Stem Cells in Arthritis

**FIGURE 2:**
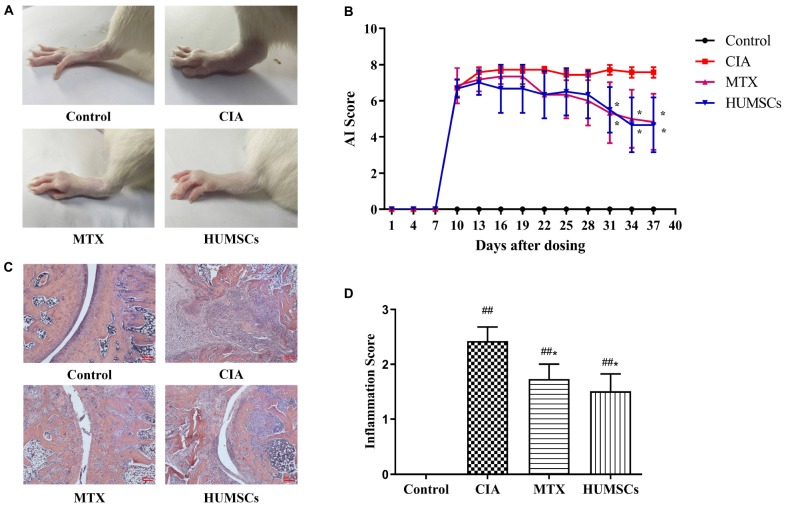
Therapeutic effect of human umbilical mesenchymal stem cells (HUMSCs) transplantation on the rat model of collagen-induced arthritis (CIA). The rats were injected with HUMSCs *via* the tail vein (1 × 10^6^ cells/rat, only once) or orally administered methotrexate (MTX; 1.5 mg/kg, twice a week) or pure water (both the control and CIA groups) for 28 days beginning on day 10 after primary immunization, and the arthritic index (AI) and inflammation score were then evaluated. **(A)** Representative gross lesions of the ankle joints after model development and treatments. **(B)** The arthritis severity was assessed every 3 days. Line plots show the AI score of all the groups. **(C)** Representative pathological sections of the ankle joints subjected to H&E staining. Scale bar = 200 μm. **(D)** The degree of inflammation in the ankle joints was scored according to lymphocyte infiltration, synovial hyperplasia, pannus, cartilage damage, and joint destruction based on H&E staining *N* = 8. The data are presented as the mean ± *SEM*. ^##^*P* < 0.01 compared with the Control group, *P < 0.05 compared with the CIA group. Control group = normal control group, CIA group = model group, MTX treatment group = positive control group, HUMSCs group = HUMSCs transplantation group.

On day 38, the CIA group exhibited severe swelling and degeneration of the ankle joints, whereas these effects were alleviated 28 days after HUMSC transplantation (Figure 2A). In addition, the AI of the MTX and HUMSCs groups decreased over time compared with the CIA group, and the difference was statistically significant starting at day 31 (*P* < 0.05) (Figure 2B). H&E staining and inflammation score revealed severe pathological changes, including lymphocyte infiltration, synovial hyperplasia, cartilage damage, joint destruction, and excessive formation of pannus in the ankles of the rats belonging to the CIA group, and MTX and HUMSC treatment of rats with CIA significantly reduced CIA joint injury and inflammation (*P* < 0.05) (Figures 2C,D). These findings illustrated that HUMSCs arrested the development and progression of CIA in rats.

### Human Umbilical Mesenchymal Stem Cells Tissue Localization in Human Umbilical Mesenchymal Stem Cell-Transplanted Rats

**FIGURE 3:**
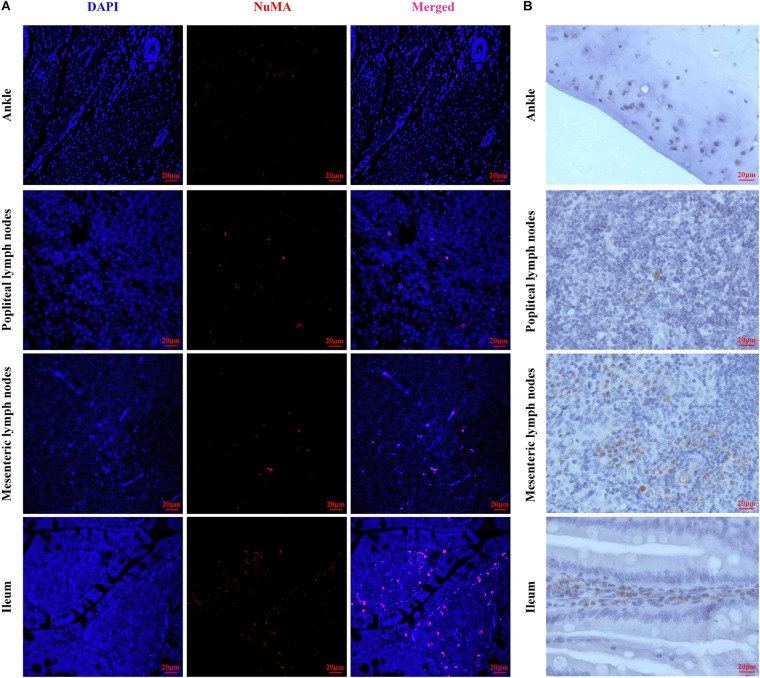
Representative images of human umbilical mesenchymal stem cells (HUMSCs) in the different tissues examined. **(A)** Representative immunofluorescence images of NuMA protein expression in the ankle, popliteal lymph node (PLN), mesenteric lymph node (MLN), and ileum. **(B)** Representative immunohistochemical images of NuMA protein expression in the ankle, PLN, MLN, and ileum (magnification, 20×).

To observe the homing of HUMSCs in the CIA rats, NuMA, a human cell-specific nuclear antigen, was used for the detection of HUMSCs in the PLN, MLN, ileum, and knee joints by IF and IHC. As shown in Figures 3A,B, extremely small numbers of protein-positive cells were found in all the observed tissues, which demonstrated that HUMSCs homed to the PLN, MLN, ankle cartilage, and ileum mucosa.

### Human Umbilical Mesenchymal Stem Cells Alleviated Bone Erosion and Bone Destruction in Arthritis

**FIGURE 4:**
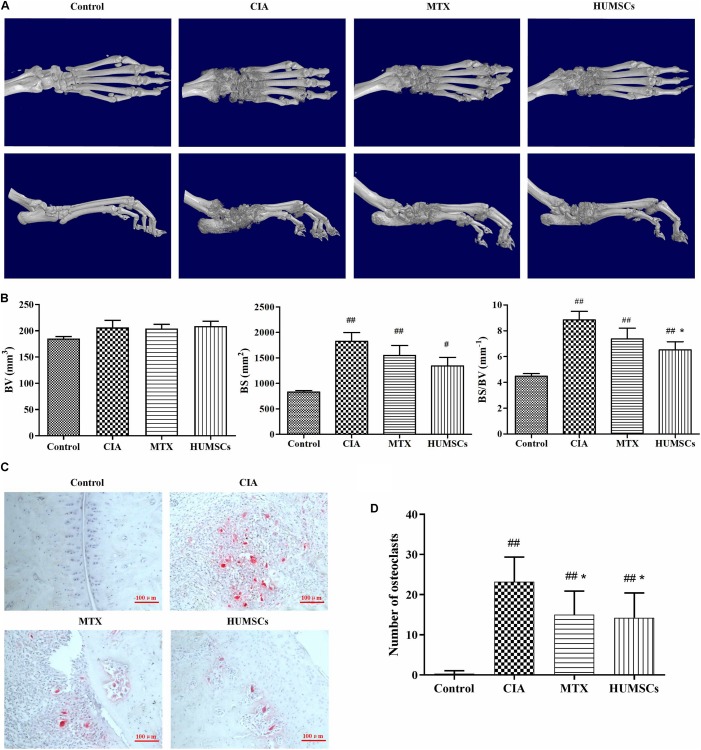
Bone-protective effect of human umbilical mesenchymal stem cells (HUMSCs) transplantation in the ankle joints of rats with collagen-induced arthritis (CIA). Three-dimensional images of the left ankle joints and paws were reconstructed and analyzed based on micro-CT scans. The osteoclasts in the ankle joints were detected and counted after tartrate-resistant acid phosphatase (TRACP) staining. **(A)** Representative three-dimensional (3D) images of the ankle joints and paws taken from above and the side. **(B)** Bar plots showing bone volume (BV), bone surface (BS), and ratio of bone surface to bone volume (BS/BV). **(C)** Representative images of ankle joints osteoclasts detected by TRACP staining. **(D)** Bar plots showing the mean numbers of osteoclasts in the ankle joints. The data are presented as the mean ± *SEM N* = 8. ^#^*P* < 0.05 compared with the Control group, ^##^*P* < 0.01 compared with the Control group, *P < 0.05 compared with the CIA group.

The ankle joints were reconstructed in 3D and are shown in Figure 4A. CIA caused simultaneous and severe bone erosion of the ankle, digital joints, and metatarsal joints, and treatment with MTX and HUMSCs transplantation significantly decreased the degree of bone erosion in these joints. Furthermore, the BV, BS, and BS/BV were analyzed for the quantitative evaluation of bone destruction (Figure 4B). The BV did not differ among the Control, CIA, MTX, and HUMSC groups. In addition, the BS was obviously increased in the CIA compared with the Control group (*P* < 0.01), but MTX and HUMSCs had no effect on the BS. However, HUMSC transplantation significantly reduced the BS/BV (*P* < 0.05). The differentiation and function of OCs play a key role in bone erosion and bone destruction in both CIA and RA (Zhao et al., 2018). To determine whether the bone-protective effect of HUMSCs on joints was related to OC inhibition, TRACP staining was performed, and the ankle OCs were counted (Figures 4C,D). As expected, almost no OCs were found in the ankle joints in the Control group, whereas a large number of OCs were observed in the ankle joints of the CIA group. In addition, both MTX treatment and HUMSC transplantation significantly reduced the number of OCs in the ankle joints compared with those found in the CIA group (*P* < 0.05). These results suggested that the HUMSC-mediated improvement in CIA is related to inhibition of the differentiation of OCs to protect bone.

### Human Umbilical Mesenchymal Stem Cells Regulated Immune Status in the Popliteal Lymph Node

**FIGURE 5:**
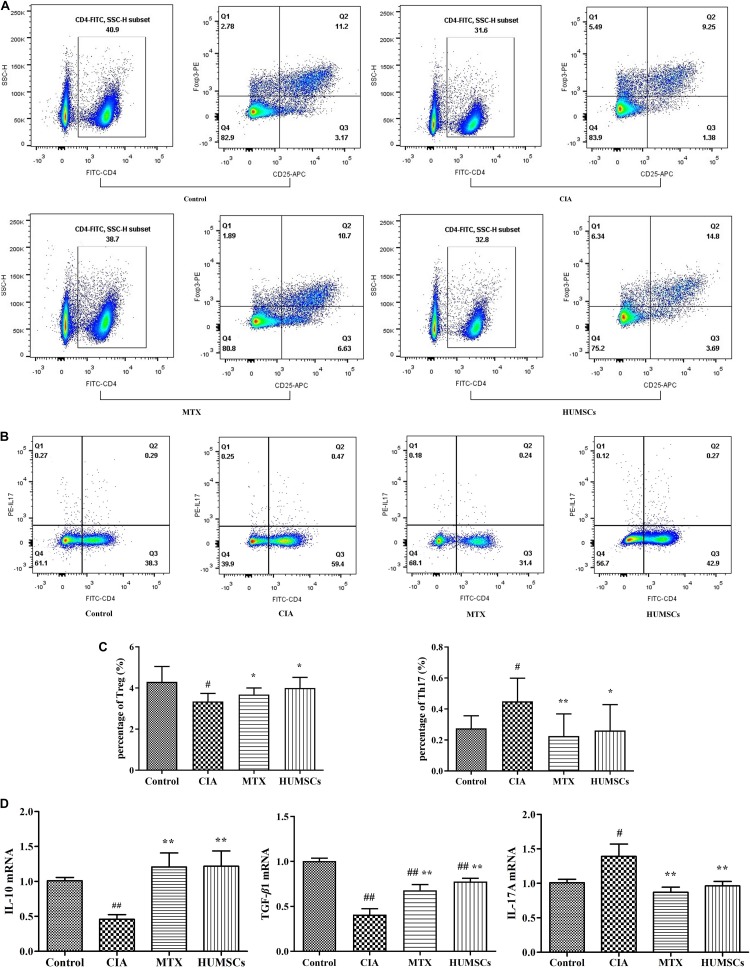
Human umbilical mesenchymal stem cells (HUMSCs) transplantation-mediated regulation of the percentage of T regulatory cells (Tregs) and T helper (Th)17 cells and related gene expression in the popliteal lymph node (PLN) of rats with collagen-induced arthritis (CIA). The percentages of Tregs and Th17 cells were detected by flow cytometry, and the RNA expression levels of interleukin (IL)-10, transforming growth factor (TGF)-β1, and IL-17A were determined by quantitative real-time PCR (Q-PCR). **(A,B)** Representative flow cytometry images of Tregs and Th17 cells in the PLN. **(C)** Bar plots showing the percentages of Tregs and Th17 cells. **(D)** Bar plots showing the mean relative RNA expression levels of IL-10, TGF-β1, and IL-17A. The data are presented as the mean ± *SEM N* = 8. ^#^*P* < 0.05 compared with the Control group, ^##^*P* < 0.01 compared with the Control group, *P < 0.05 compared with the CIA group, **P < 0.01 compared with the CIA group.

The immune status in the PLN was used to represent that of the lesion site as the PLN was the closest immune tissue to the lesion site. The percentages of Tregs and Th17 cells in the PLN were determined *via* flow cytometry (Figures 5A,B). The percentage of Tregs in the CIA group was lower than that in the Control group (*P* < 0.05). In contrast, the percentage of Tregs was higher in the HUMSC and MTX groups than in the CIA group (*P* < 0.05). The percentage of Th17 cells was higher in the CIA group than in the Control group (*P* < 0.05), while the percentage of Th17 cells in both the HUMSC and MTX groups was decreased compared to that in the CIA group (*P* < 0.01, *P* < 0.05; Figure 5C). Additionally, as shown in Figure 5D, the mRNA expression of IL-10, TGF-β1, and IL-17A in the PLN was regulated by HUMSCs and MTX. The levels of both IL-10 and TGF-β1 in the CIA group were lower than those in the Control group, while the level of IL-17A was higher (*P* < 0.01, *P* < 0.05, respectively). HUMSCs and MTX enhanced the mRNA expression of IL-10 and TGF-β1 and reduced the level of IL-17A compared to those in the CIA group (*P* < 0.05). The results indicated that HUMSCs and MTX regulated the immune status and function of Tregs and Th17 cells.

### Human Umbilical Mesenchymal Stem Cells Regulated the Expression of Serum Cytokines

**FIGURE 6:**
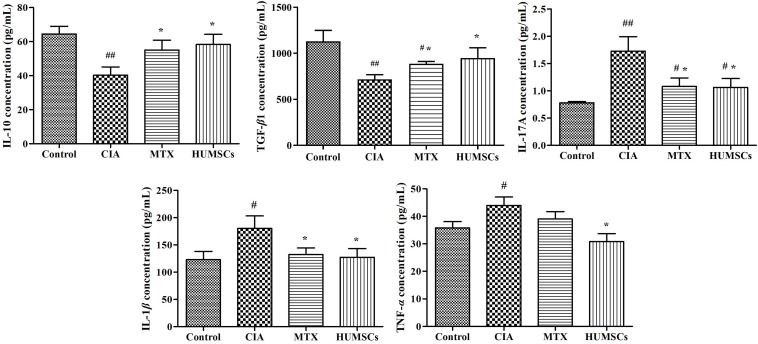
Human umbilical mesenchymal stem cells (HUMSCs) transplantation-mediated regulation of cytokines in the collagen-induced arthritis (CIA) rats. The serum cytokine levels were measured by Luminex assays. Bar plots showing the mean levels of interleukin (IL)-10, transforming growth factor (TGF)-β1, IL-17A, IL-1β, and tumor necrosis factor (TNF)-α in all the groups *N* = 8. The data are presented as the mean ± *SEM*. ^#^*P* < 0.05 compared with the Control group, ^##^*P* < 0.01 compared with the Control group, *P < 0.05 compared with the CIA group.

As shown in Figure 6, the CIA group exhibited downregulated expression of serum IL-10 and TGF-β*1* and upregulated levels of IL-17A, IL-1β, and TNF-α compared with the Control group (*P* < 0.05, *P* < 0.01, respectively). HUMSC transplantation increased the levels of IL-10 and TGF-β*1* and decreased the levels of IL-17A, IL-1β, and TNF-α compared with those found in the CIA group (*P* < 0.05). MTX also upregulated the expression of IL-10 and TGF-β*1* (*P* < 0.05) and downregulated the expression of IL-17A and IL-1β compared with those in the CIA group (*P* < 0.05). The MTX group also showed decreased TNF-α expression compared with the CIA group, but this difference was not statistically significant. The results showed that HUMSCs can regulate not only the local but also the systemic immune state.

### Human Umbilical Mesenchymal Stem Cells Modulated the Immune Status of Gut-Associated Lymphoid Tissues

To explore the effect of HUMSCs on the MLN and ileum, the percentages of Tregs and Th17 and B cells in the MLN and PP and among LPL were detected by flow cytometry (Table 2). CIA increased the percentage of Th17 cells in the MLN (*P* < 0.05). MTX treatment increased the percentage of only Tregs (*P* < 0.05), whereas HUMSC transplantation both increased the percentage of Tregs and decreased the percentage of Th17 cells (*P* < 0.05). Modeling and treatment did not appear to affect B cells. The proportions of the three cell subtypes in PP changed slightly with treatment, and no difference in the proportion of Tregs between the Control and CIA groups. Interestingly, the proportion of Tregs in MTX group was lower than those in the Control and CIA groups (*P* < 0.05), whereas the proportion of Tregs in the HUMSC group was higher than that in the CIA group (*P* < 0.05) and even higher than that in the Control group (*P* < 0.01). The change in the proportion of Th17 cells in PP was similar to that in the MLN, but MTX intervention also reduced the proportion of Th17 cells in PP (*P* < 0.05). Although the proportion of B cells in the MLN was not significantly different, the proportion of B cells in PP decreased after modeling (*P* < 0.05), and the proportions of B cells in the MTX and HUMSC groups increased after treatment and were higher than those in the rats belonging to the Control group (*P* < 0.05). The proportions of Tregs and Th17 cells among LPL were higher than those in the MLN and PP. The assessment of the variations in Tregs among LPL between the groups revealed a trend similar to that found in the MLN, whereas the variations in Th17 cells and B cells followed a trend similar to those found for Th17 cells and B cells in PP, respectively, with the only difference being that the proportion of B cells after treatment was between the proportions of B cells in the Control and CIA groups. The above-described results suggested that HUMSC therapy exerts extensive immunomodulatory effects on GALTs. The most affected tissues were those closest to the intestinal contents, and these effects might be induced by metabolites or antigens related to the gut microbiota.

**TABLE 2:** Percentages of Tregs, Th17, and B cells in gut-associated lymphoid tissues (*N* = 8).

Tissues	Group	Cell type		
		Tregs (%)	Th17 cells (%)	B cells (%)
Mesenteric lymph node	Control	6.07 ± 2.21	0.20 ± 0.12	31.50 ± 3.34
	CIA	5.41 ± 1.31	0.34 ± 0.05^#^	32.82 ± 4.18
	MTX	7.06 ± 0.80*	0.32 ± 0.08	34.34 ± 3.73
	HUMSCs	7.27 ± 0.73*	0.24 ± 0.05*	35.20 ± 5.43
Peyer’s patch	Control	10.77 ± 1.62	0.67 ± 0.39	44.80 ± 5.74
	CIA	10.86 ± 1.79	1.17 ± 0.90^#^	39.23 ± 4.81^#^
	MTX	8.29 ± 1.90^#^*	0.46 ± 0.27*	55.06 ± 4.63^#^**
	HUMSCs	14.81 ± 3.09^##^*	0.48 ± 0.18*	54.12 ± 6.82^#^**
Lamina propria lymphocyte	Control	27.98 ± 2.89	2.57 ± 1.46	37.37 ± 2.96
	CIA	21.03 ± 5.41^#^	8.30 ± 2.02^#^	31.92 ± 3.85^#^
	MTX	31.08 ± 9.66*	3.46 ± 0.89**	46.26 ± 11.39*
	HUMSCs	28.75 ± 4.15*	3.04 ± 1.42**	49.00 ± 15.89*

The data are presented as the mean ± *SEM*. ^#^*P* < 0.05 compared with the Control group, ^##^*P* < 0.01 compared with the Control group, *P < 0.05 compared with the CIA group, **P < 0.01 compared with the CIA group. Tregs, T regulatory cells; Th17, T helper 17; CIA, collagen-induced arthritis; MTX, methotrexate; HUMSCs, human umbilical mesenchymal stem cells.

**FIGURE 7:**
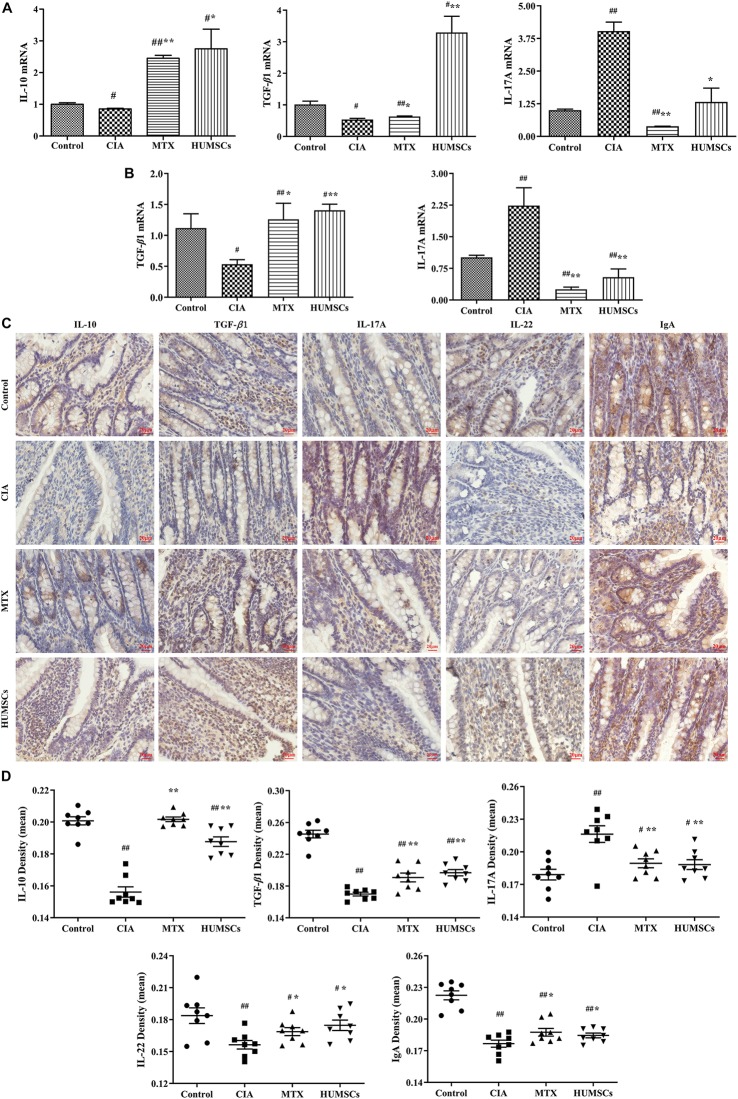
Human umbilical mesenchymal stem cells (HUMSCs) modulate the immune status of the mesenteric lymph node (MLN) and ileum. The interleukin (IL)-10, IL-17A, and transforming growth factor (TGF)-β*1* mRNA expression levels in the MLN and the IL-17A and TGF-β*1* mRNA expression levels in the ileum were detected by quantitative real-time PCR (Q-PCR). The protein expression of IL-10, IL-17A, TGF-β*1*, IL-22, and immunoglobulin A (IgA) was determined by immunohistochemistry (IHC) assay. **(A)** Gene expression of IL-10, IL-17A, and TGF-β*1* in the MLN. **(B)** Gene expression of IL-17A and TGF-β*1* in the ileum. **(C)** Representative images of the ileum obtained by IHC (20×). **(D)** Protein expression of IL-10, IL-17A, TGF-β*1*, IL-22, and IgA in the ileum *N* = 8. The data are presented as the mean ± *SEM*. ^#^*P* < 0.05 compared with the Control group, ^##^*P* < 0.01 compared with the Control group, *P < 0.05 compared with the CIA group, **P < 0.01 compared with the CIA group.

Furthermore, various gene and protein expression levels were detected by Q-PCR and IHC, respectively (Figure 7). Modeling significantly decreased the IL-10 levels in the MLN and the TGF-β1 levels in the MLN and ileum (*P* < 0.05), and treatment with MTX and HUMSC transplantation increased the expression of IL-10 and TGF-β1 to levels that were higher than that those found in the Control group (*P* < 0.05 and *P* < 0.01, respectively) (Figures 7A,B). The expression levels of IL-17A in the MLN and ileum were similar. The IL-17A gene expression levels were highest in the CIA group and decreased following intervention with MTX and HUMSCs (*P* < 0.05 and *P* < 0.01, respectively), and the lowest IL-17A gene level was found in the MTX group (Figures 7A,B). The *in situ* protein expression levels were similar to the corresponding gene expression levels (Figures 7C,D). Downregulated IL-10 and TGF-β1 expression and upregulated IL-17A expression were detected in the ileum of rats with CIA, and as expected, both MTX treatment and HUMSC transplantation increased the levels of IL-10 and TGF-β1 and decreased the expression of IL-17A (*P* < 0.05 and *P* < 0.01, respectively). The above-described results indicated that the levels of the major cytokines of Tregs and Th17 cells were abnormal in the MLN and ileum of rats with CIA and that MTX and HUMSCs effectively reversed Tregs and Th17 cell dysfunction. These results suggested that HUMSCs regulate the proportions and functions of Tregs and Th17 and B cells in GALTs.

We also tested the expression of IL-22 and IgA because IL-22 plays an important role in resisting pathogens and repairing the intestinal barrier by inducing intestinal epithelial cells (IECs) to produce antimicrobial peptides, and secretory IgA (SIgA) is known to regulate intestinal microorganisms. The IL-22 and IgA levels in the CIA group were significantly lower than those in the Control group (*P* < 0.01), which suggested that CIA modeling affects the mucosal immune barrier in the ileum to a certain extent and decreased the clearance of pathogenic microorganisms. MTX treatment and HUMSC transplantation affected the IL-22 and IgA levels (*P* < 0.05, *P* < 0.01, respectively) (Figures 7C,D), which suggested that both treatments can reshape the immune barrier and restore the regulatory effect of the intestinal tract on intestinal bacteria.

### Human Umbilical Mesenchymal Stem Cells Regulated the Intestinal Microbial Community in Rats With Collagen-Induced Arthritis

**FIGURE 8:**
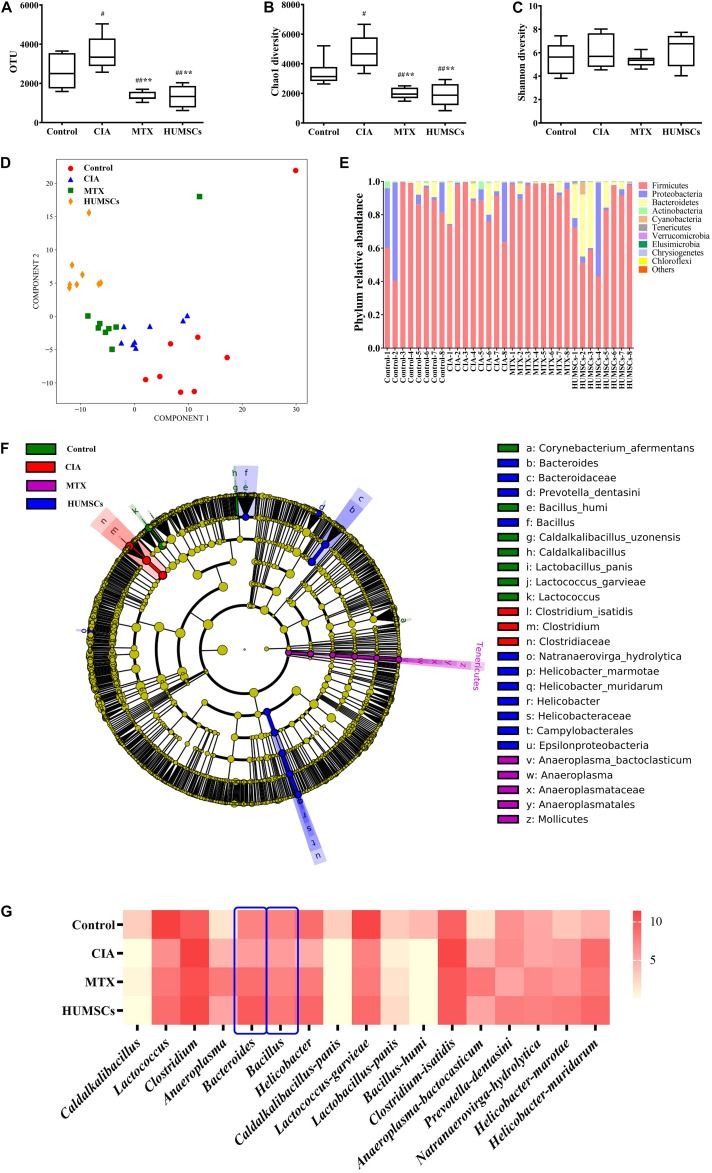
Changes in the gut microbiota in collagen-induced arthritis (CIA), methotrexate (MTX), and human umbilical mesenchymal stem cells (HUMSCs) groups. The alpha diversity, beta diversity, and the typical bacterial genera and species were obtained by 16S sequencing. **(A–C)** Bar plots showing the mean Chao1 and Shannon diversity indices and the operational taxonomic units (OTUs) of all groups. **(D)** Relative abundance of the top 10 most abundant phyla in each sample. **(E)** The differences in the intestinal microbial communities among the groups were assessed by partial least square discriminant analysis (PLS-DA). **(F)** Taxonomical differences among the groups at the different levels. The dominant bacteria were determined by Lefse analyses. **(G)** Relative abundances among the groups at the genus and species levels *N* = 8. The data in panels **(A–C)** are presented as the medians [interquartile range (IQR)]. ^#^*P* < 0.05 compared with the Control group, ^##^*P* < 0.01 compared with the Control group, *P < 0.05 compared with the CIA group, **P < 0.01 compared with the CIA group.

The intestinal microbial diversity of the ileum was measured by determining the OTUs and the Chao1 and Shannon diversity indices (Figures 8A–C). The OTUs and Chao1 diversity indices in the CIA group were higher than those in the Control group (*P* < 0.05), and both of these measures were lower in the MTX and HUMSCs groups compared with the Control and CIA groups (*P* < 0.01). The analysis of the Shannon diversity index revealed no significant difference among the four groups. The results shown in Figures 8A–C indicated that CIA modeling and both treatments changed the rats’ intestinal microbial species and the community richness but had no effect on the community diversity.

The similarity between the intestinal microbial communities was analyzed by PLS-DA. As shown in Figure 8D, the bacterial structures of the Control, CIA, MTX, and HUMSC groups were distributed in a counterclockwise direction. With the exception of a few outliers, the microflora structures in each group were relatively similar, and the difference between the HUMSC rats and normal rats, between the HUMSC rats and CIA rats were the obvious, but the microbial communities of the CIA group were very similar to those of the MTX group. The results indicated that the therapeutic effect of MTX on CIA might only be slightly related to the gut microbiota, whereas the effect of HUMSCs might be largely mediated by the gut microbiota.

An overview of the gut microbiota in the four groups at the phylum level is shown in Figure 8E. Overall, the bacteria with the highest abundance belonged to the phylum *Firmicutes*, followed by the phyla *Proteobacteria* and *Bacteroidetes*. The abundances of the phyla *Proteobacteria* and *Bacteroidetes* among the rats varied considerably. The phyla *Chrysiogenetes* and *Chloroflexi* disappeared after treatment with MTX and HUMSCs.

The intestinal microbial species in the different groups were compared by Lefse analysis to characterize the changes in the intestinal microflora structure (Figure 8F). The relative abundances of two genera (*Caldalkalibacillus* and *Lactococcus*) and five species (*Caldalkalibacillus panis*, *Lactococcus garvieae*, *Lactobacillus panis, Bacillus humi*, and *Corynebacterium afermentans*) were highest in the control group. The dominant bacteria in the CIA group were relatively unitary: family *Clostridiaceae*, genus *Clostridium*, and species *Clostridium isatidis.* After MTX intervention, the relative abundance of one unit was higher than that of the other three groups from the phylum to the species level: phylum *Tenericutes*, class *Mollicutes*, order *Anaeroplasmatales*, family *Anaeroplasmataceae*, genus *Anaeroplasma*, and species *Anaeroplasma bactocasticum.* HUMSC therapy changed the most bacterial units: one class (*Epsilonproteobacteria*), one order (*Campylobacterales*), two families (*Bacteroidaceae* and *Helicobacteraceae*), three genera (*Bacteroides, Bacillus*, and *Helicobacter*), and four species (*Prevotella dentasini*, *Natranaerovirga hydrolytica*, *Helicobacter marotae*, and *Helicobacter muridarum*). The abovementioned results indicated that CIA modeling plays an important role in the changes in the gut microbiota and that HUMSCs had a greater influence on the flora than MTX.

The specific gut microbiota changes were further assessed at the genus and species levels (Figure 8G). CIA modeling significantly decreased the abundances of various genera, including *Caldalkalibacillus*, *Lactococcus*, *Bacteroides*, *Bacillus*, and others and various species, including *Caldalkalibacillus panis*, *Lactococcus garvieae*, *Lactobacillus panis*, and *Bacillus humi* and others, and treatment with MTX and HUMSCs increased the abundances of the genera *Lactococcus*, *Bacteroides*, and *Bacillus* and the species *Lactococcus garvieae* and *Lactobacillus panis* but did not affect the abundances of the genus *Caldalkalibacillus* and the species *Caldalkalibacillus panis* and *Bacillus humi*. Conversely, the relative abundance of the genus *Clostridium* and the species *Clostridium isatidis*, *Anaeroplasma bactocasticum*, *Helicobacter marotae*, and *Helicobacter muridarum* increased after modeling. In addition, treatment with MTX and/or HUMSCs slightly decreased the abundances of some genera and species (genus *Clostridium* and species *Clostridium isatidis* and *Helicobacter muridarum*) but increased the abundances of some bacteria (species *Anaeroplasma bactocasticum* and *Helicobacter marotae*). In addition, a few bacteria were not affected by CIA modeling but exhibited an altered abundance after treatment with MTX and HUMSCs (species *Prevotella dentasini* and *Natranaerovirga hydrolytica*). Collectively, these data indicate that although the major intestinal microbial communities exhibited differences after MTX and HUMSC treatment, the abundance of some bacteria still changed in a synchronous manner. These results indicated that MTX and HUMSCs might modulate mucosal immunity through a common mechanism involving the gut microbiota.

### Gut Microbiota Regulated the Aryl Hydrocarbon Receptor Pathway Through Indole Metabolism

**FIGURE 9:**
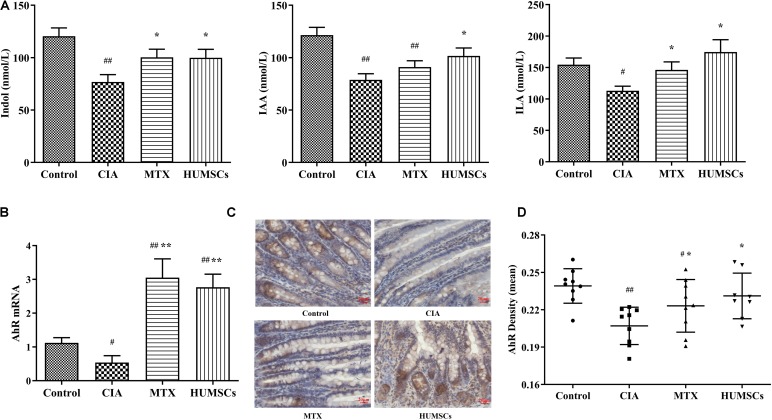
Levels of indole and its derivatives in the plasma and aryl hydrocarbon receptor (AhR) expression in the ileum. The levels of indole, indoleacetic acid (IAA), and indole-3-lactic acid (ILA) in the plasma were detected by liquid chromatography-tandem mass spectrometry (LC-MS/MS), and the mRNA and protein expression levels of AhR were determined by quantitative real-time PCR (Q-PCR) and immunohistochemistry (IHC), respectively. **(A)** Bar plots showing the mean levels of indole, IAA, and ILA in all the groups. **(B)** AhR gene expression in the ileum. **(C)** Representative images of the ileum obtained by IHC (20×). **(D)** AhR protein expression in the ileum *N* = 8. The data are presented as the mean ± *SEM*. ^#^*P* < 0.05 compared with the Control group, ^##^*P* < 0.01 compared with the Control group, *P < 0.05 compared with the CIA group, **P < 0.01 compared with the CIA group.

Based on the above-described results and those obtained in other studies, we found that *Bacteroides* and *Bacillus* can metabolize dietary tryptophan to produce indole, IAA, or ILA (Amin and Latif, 2017; Roager and Licht, 2018). Interestingly, these metabolites are AhR ligands that can stimulate the AhR and regulate intestinal mucosal immunity (Shinde and McGaha, 2018). Therefore, we detected the levels of indole, IAA, and ILA in plasma by LC-MS/MS and the AhR gene and protein expression levels in the ileum by Q-PCR and IHC, respectively. As shown in Figure 9A, the levels of indole, IAA, and ILA in the CIA group were significantly lower compared with those in the Control group (*P* < 0.05 and *P* < 0.01, respectively). Treatment with HUMSCs increased the levels of these three metabolites compared with those in the CIA group, and this difference was significant (*P* < 0.05). The levels of the three metabolites in the MTX group were also increased, but only the indole and ILA levels were different from those in the rats with CIA (*P* < 0.05). This result was consistent with the results from the analysis of the gut microbiota because the bacteria found at higher abundances in the HUMSC group (mainly *Lactococcus* and *Bacteroides*) can metabolize tryptophan and produce indole and other metabolites that are absorbed into the blood through the small intestine.

The assessment of the AhR expression in the ileum (Figures 9B–D) revealed that the AhR gene and protein expression levels in the CIA group were consistent with changes in the levels of tryptophan metabolites, which were found at lower levels in this group compared with the Control group (*P* < 0.05 and *P* < 0.01, respectively). Treatment with MTX and HUMSCs upregulated the mRNA expression of AhR by 3-fold and more than 2-fold, respectively. However, the change in AhR protein expression was relatively minor compared with the change in its gene expression, and the AhR gene expression level in the treatment groups was between those in the Control and CIA groups but significantly differed from that in the CIA group (*P* < 0.05). These results confirms that indole, IAA, and ILA produced during bacterial decomposition activate the AhR. The regulatory effect of intestinal bacteria on mucosal immunity is likely dependent on activation of the AhR, a receptor that reportedly regulates the differentiation and function of Tregs and Th17 and B cells.

## Discussion

Numerous studies have explored the therapeutic effects of HUMSCs that mediate the balance between Tregs and Th17 cells in RA (Ma et al., 2019). Several researchers have explored the effect and mechanism of other drugs on RA from the perspective of the gut microbiota (Bodkhe et al., 2019). However, no researchers have linked Tregs/Th17 cells with the gut microbiota to elucidate the therapeutic mechanism of HUMSCs. Based on the tissues to which HUMSCs home, we explored the influence of HUMSCs on bone destruction in the ankle joints and the proportions and functions of Tregs and Th17 cells in the nearest lymph node—the PLN. Furthermore, we focused on functions related to Tregs and Th17 cells and the functions through which B cells regulate the gut microbiota in GALTs: the MLN, PP, and LPL. We examined the changes in the gut microbiota in the ileum in depth and found changes in most bacteria involved in the metabolism of tryptophan to indole. To validate these results, we measured the plasma levels of indole and some of its derivatives and the AhR levels in the ileum, and the results showed a key link between indole metabolism and the immune system. Based on the current results, we can confirm that HUMSCs act as a therapeutic agent in CIA by regulating the interaction between host immunity and the gut microbiota and speculate that AhR is the key link between these factors.

Several clinical and experimental studies have investigated stem cell transplantation for the treatment of RA. A clinical trial conducted in Iran found that autologous bone marrow-derived mesenchymal stem cells ameliorated the clinical symptoms of refractory RA (Ghoryani et al., 2019). Another phase 1/2 clinical trial observed that autologous bone marrow-derived mesenchymal stromal cells increase the clinical efficacy of MTX and prednisolone and reduce their use (Shadmanfar et al., 2018). Animal experiments using relatively uniform methods have also shown that HUMSCs can be utilized to treat CIA (Cheng-Chi et al., 2012; Shin et al., 2016). Specifically, stem cells were injected at the same dose (1 × 10^6^ cells/rat or mouse) in most studies. However, the graft sites differed (tail vein, abdominal cavity, and articular cavity), and the observation time varied from half a month to a month. Our purpose was to observe the systemic regulatory effect of HUMSCs, and based on the treatment duration used in a previous experiment (Zhao et al., 2018), 1 × 10^6^ cells/rat were injected *via* the tail vein for one month. Some researchers have tracked the entry of stem cells into the body. Liu et al. (2010) showed that intraperitoneally injected HUMSCs could not be detected in joints, but a positive signal for HUMSCs was found in the spleen on days 3 and 7 and disappeared on day 14 after injection. In addition, Wu C. C. et al. (2016) detected the expression of HLA-A protein, which is characteristic of HUMSCs, in knee cartilage and synovial tissue on day 14 after the injection of HUMSCs into the joint. In our study, positive NuMA signaling was simultaneously detected in the knee, PLN, MLN, and ileum mucosa on day 28 after HUMSC injection. The differences in the above-described results might be due to the different transplantation methods and transplantation durations used.

Because Tregs are immunosuppressive cells, their proportion and function are crucial in the suppression of ADs. Tregs act as cytotoxic agents through cell–cell interactions and function by secreting the cytokines IL-10 and TGF-β1 (Shevach, 2009). In the intestine, Tregs play an important role in the maintenance of immune tolerance to dietary antigens and the gut microbiota (Honda and Littman, 2016). Unlike Tregs, another cell subgroup, Th17 cells, induces immune activation by secreting IL-17A to promote inflammation (Patel and Kuchroo, 2015). However, the production of IL-17A and IL-22 by Th17 cells in the gut contributes to the defense against intestinal pathogens (Ubeda and Pamer, 2012). Tregs/Th17 cells are the most studied immune cells in the peripheral blood and spleen; although the immune statuses in these two tissues reflect the immune state of the system, they are not typical (Liu et al., 2010; Ma et al., 2019). More detailed studies of this mechanism have targeted tissues to reveal the local immune status, such as targeting Tregs/Th17 cells to the MLN and PP (Tong et al., 2015). However, the involvement of HUMSCs in influencing Tregs/Th17 cells in the PLN and among LPL in CIA has not been reported. We identified the distribution of HUMSCs in CIA through their localization and then specifically determined the proportions of these two cell subtypes in HUMSC-positive tissues, namely, the PLN, MLN, PP, and LPL. In addition, SIgA plays an important role in the resistance to intestinal bacterial adhesion and endotoxin neutralization (Iijima et al., 2001). Because precursor cells secrete SIgA, we also measured the proportion of B cells in the above-listed GALTs (Reboldi et al., 2016). Our results are generally consistent with those obtained in the limited number of previous reports. HUMSC therapy significantly increased the proportion of Tregs; significantly decreased the proportion of Th17 cells in the PLN, MLN, and PP and among the LPL compared with that found in the CIA group; and significantly increased the proportion of B cells in PP and among the LPL. The abovementioned results suggested that the immune status of intestinal-associated lymphoid tissues worsened after modeling and that the homing of HUMSCs partially reversed immune disorders, and the detection of Treg-, Th17 cell-, and B cell-related factors verified the above-described results.

The importance of the gut microbiome in RA has been reported in both human patients and mouse models. Alterations to the gut microbiota are related to the risk and severity of RA, and probiotic transplantation is beneficial for RA remission. Toivanen et al. (2002) showed that patients with early RA had on average only half as many bacteria belonging to the genera *Bacteroides*, *Prevotella*, and *Porphyromonas* compared with control subjects. Another study conducted by Vaahtovuo et al. (2008) similarly found that RA patients were characterized by a different fecal diversity with a decreased abundance of bacteria belonging to the *Bacteroides–Porphyromonas–Prevotella* group, the *Bacteroides fragilis* subgroup, and the *Eubacterium rectale–Clostridium coccoides* group compared with patients with fibromyalgia. *Lactobacillus casei* has been shown to be an effective probiotic in the treatment of RA. Alipour et al. (2014) treated 22 subjects with *L. casei* and compared them with 24 subjects treated with placebo. The C-reactive protein (CRP) level, number of inflamed and painful joints, and 28-joint disease activity score (DAS-28) were significantly decreased in the 22 subjects compared with the 24 controls. In animal models, both intestinal bacteria known to be correlated with arthritis and differences in the composition of the gut microbiota between animals with CIA and normal animals and even between arthritis-susceptible and healthy or arthritic hosts were consistently detected. In the K/BxN mouse, gut-residing segmented filamentous bacteria (SFB) were found to drive the rapid development of destructive arthritis (Doube and Collins, 1988). Liu et al. (2016) revealed that the genus *Lactobacillus* was the dominant genus in mice susceptible to CIA prior to the onset of arthritis. The development of the disease increased the abundances of the families *Bacteroidaceae*, *Lachnospiraceae*, and *S24-7* in CIA-susceptible mice. Notably, germ-free mice treated with the microbiota of CIA-susceptible mice showed a higher frequency of arthritis induction than those treated with the microbiota of CIA-resistant mice. Our results showed that CIA modeling decreased the abundances of the genera *Bacteroides, Lactococcus*, and species *Lactococcus garvieae* compared with those in the Control group, which was partly consistent with the above-described findings.

The gut serves as a bridge between intestinal microorganisms and their host. Intestinal bacteria synthesize and alter multiple compounds that impact physiology and immunity (Rooks and Garrett, 2016). Numerous studies have shown that Tregs and Th17 cells are simultaneously the target cells of intestinal commensal microbiota and the key to activating the host defense against pathogens, which could result in the avoidance of other immunopathologic consequences (Bennike et al., 2017). Under conventional conditions, *Lactobacillus bifidus* induces arthritis in IL-1ra^–/–^ mice, which exhibit a decreased number of Tregs and an increased number of Th17 cells (Abdollahi-Roodsaz et al., 2008). In contrast, *Bifidobacterium infantis* can induce Tregs to play an anti-inflammatory role, and *B. fragilis* polysaccharide stimulates Tregs to produce IL-10 (Abdollahi-Roodsaz et al., 2008). A study conducted by Zhang et al. (2016) revealed that the supplementation of broiler diets with *Lactococcus garvieae B301* increased the body weight and exerted a positive effect on the serum levels of immune globulins. In addition, Wang et al. (2019) showed that recombinant *Bacillus subtilis* induces a mucosal SIgA response to pseudorabies and regulates the CD3^+^CD4^+^ T cell proliferative response by IL-10. Another study showed that a mixture of two *Clostridium* strains significantly reduces the Th17/Th1 balance (Lopez et al., 2016), and infection with *Clostridium perfringens* or *Clostridium difficile* induces a pro-inflammatory response related to Th17 cells (Yacyshyn et al., 2014; Yu et al., 2017). In the current study, regarding the relative abundances of the genera *Lactococcus*, *Bacillus*, and *Bacteroides* and the species *Lactococcus garvieae*, Tregs and the related cytokines and SIgA were increased in both MTX- and HUMSC-treated rats, whereas regarding the relative abundances of the genus *Clostridium*, Th17 cells and related cytokines were upregulated in rats with CIA and reduced after the treatments, as expected. Based on the discussion and results described above, we speculated that changes in the gut microbiota caused changes in immune regulation.

A review of the literature revealed that the bacterial genera *Bacillus* and *Bacteroides* are involved in the metabolism of tryptophan into indole and its derivatives (Shao et al., 2015; Roager and Licht, 2018). Interestingly, the components regulated by these bacteria, namely, indole, IAA, and ILA, are ligands of the AhR (Gao et al., 2018). The available evidence suggests that the AhR is a key sensor that allows immune cells to adapt to environmental conditions and that the inhibition of AhR activity is associated with ADs. Numerous studies have shown that activation of the AhR increases the differentiation and function of Tregs but inhibited inflammatory cytokines (Shinde and McGaha, 2018). Researchers have reported that sinomenine induces the generation of intestinal Tregs and attenuates arthritis *via* activation of the AhR even in CIA (Tong et al., 2016a). Some evidence suggests that AhR activation increases Th17 cell differentiation and the secretion of IL-17A and IL-22 (Shinde and McGaha, 2018). However, the results obtained after treatment of the CIA model consistently suggested that activation of the AhR inhibits the differentiation and function of Th17 cells (Tong et al., 2015, 2016b). This difference in results might be due to the type of ligand and animal species used and the disease type examined. In addition, Villa et al. (2017) demonstrated that intervention with 6-formylindolo[3,2-b]carbazole (FICZ), a natural agonist, increased AhR expression during B cell differentiation and the amount of AhR localized to the nucleus and significantly upregulated the expression of the downstream target gene CYP1A1 on B cells. These results imply that the AhR is involved in B cell proliferation and function. Our results demonstrated that the increased abundance of the genera *Bacteroides* and *Bacillus* might lead to increased levels of indole and its derivatives in the plasma, and these increases enhance AhR gene and protein expression in the ileum. Furthermore, activated AhR might regulate the gut microbiota by regulating Tregs and Th17 and B cells *via* a feedback loop. Although a few *Clostridium* strains, such as *Clostridium difficile* and *Clostridium sordellii*, can produce indole, IAA, or ILA (Roager and Licht, 2018), pathogenic bacteria species belonging to the genus *Clostridium* can produce toxins that induce clostridial disease. Our results showed that the genus *Clostridium* exhibited an increasing trend in the CIA group, which was in line with the function of this strain.

**FIGURE 10:**
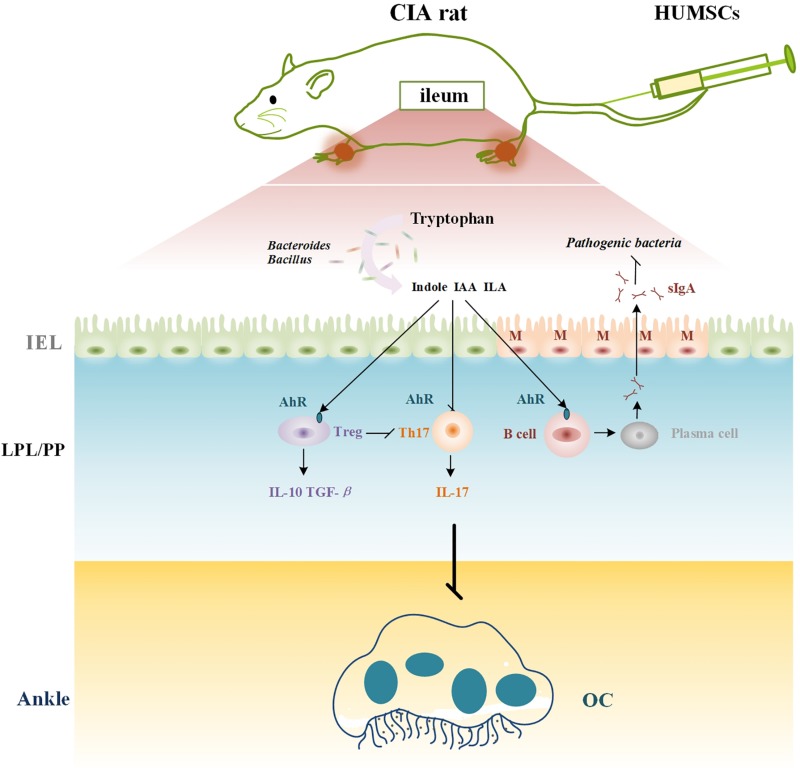
Mechanism of HUMSCs therapy in CIA rat. CIA, collagen-induced arthritis; HUMSCs, Human umbilical mesenchymal stem cells; IAA, indoleacetic acid; ILA, indole-3-lactic acid; SIgA, secretory immunoglobulin A; MLN, mesenteric lymph node; AhR, aryl hydrocarbon receptor; Treg, T regulatory cell; Th17, T helper 17 cell; OC, osteoclast; PLN, popliteal lymph node.

In summary, our results suggest that HUMSCs play a therapeutic role in CIA because these cells regulate immunity in rats with CIA and particularly immunity in GALTs. The underlying mechanism might be related to the crosstalk between immunity and the gut microbiota regulated by the AhR (Figure 10).

## Data Availability Statement

The datasets generated in this study are available from the corresponding author upon request. 16S sequencing data was uploaded to NCBI SRA with accession number PRJNA605946.

## Ethics Statement

The study involving human participants was reviewed and approved by the Research Ethics Committee at the China-Japan Friendship Hospital (2019-124-K86). The patients/participants provided their written informed consent to participate in this study. The animal study was reviewed and approved by the Institute of Clinical Medical Sciences, China-Japan Friendship Hospital, Beijing, China (No. 180207).

## Author Contributions

CX and HL designed the conceptual framework of the study. XLi and CL designed the experiments and wrote the manuscript. XLi, CL, XLu, DF, YX, and JL performed the experiments. HZ, HX, and YZ analyzed the data.

## Conflict of Interest

The authors declare that the research was conducted in the absence of any commercial or financial relationships that could be construed as a potential conflict of interest.
